# Suicide by persons with foreign background in Japan

**DOI:** 10.1371/journal.pone.0211867

**Published:** 2019-02-06

**Authors:** Michiko Ueda, Kanako Yoshikawa, Tetsuya Matsubayashi

**Affiliations:** 1 Faculty of Political Science and Economics, Waseda University, Shinjuku, Tokyo, Japan; 2 Osaka School of International Public Policy, Osaka University, Toyonaka, Osaka, Japan; Sogang University (South Korea), REPUBLIC OF KOREA

## Abstract

**Purpose:**

This study examined trends in the suicide rates of persons with foreign background in Japan.

**Methods:**

Using the nationwide death records in the Vital Statistics of Japan, we first reported the age-adjusted suicide rates of 8 foreign nationals (Brazil, China, Korea, Peru, the Philippines, Thailand, the United Kingdom, and the United States) in Japan by sex from 1980 to the mid-2010s. We also computed rate ratios to compare the suicide rate of each group with those of the Japanese population. Second, we focused on Koreans, who had the highest suicide rate in Japan. We compared the suicide rates of Koreans in Japan with Koreans in South Korea to examine whether their suicide rates were more closely related to those of their country of origin or those of their host country.

**Results:**

We found that the suicide rates of Koreans and Chinese in Japan were similar to or higher than those of Japanese, while other groups tended to show lower suicide rates. Most notably, Koreans displayed consistently high suicide rates from 1980 to the mid-2010s, which were nearly twice as high as those of the Japanese population. Korean males and females in Japan had higher suicide rates than those in South Korea.

**Conclusions:**

Immigrants in Japan were not necessarily influenced by the suicide rates of the host country. The high suicide rates among Korean residents in Japan might be explained by various disadvantages and adversities that they face in Japan.

## Introduction

Immigrants have become an important and growing segment of the population, accounting for almost 10 percent of the population in the OECD countries [1]. Immigrants often face challenges in the process of acculturation and adjustment to a new environment, especially when their traditional culture and the host country’s mainstream culture conflict [2,3]. Immigrants may be more susceptible to mental disorders due to traumatic events prior to immigration or face adverse circumstances upon arrival to their country of destination [4,5]. They may also struggle with low socio-economic status, poor working conditions, impairments in physical health, and psychological distress [6–8].

Past studies focused on suicide as a measure of immigrants’ well-being and compared immigrant suicide rates to those of their host country or country of origin; however, conclusions among such studies are inconsistent. One body of research has found a high degree of correspondence between immigrants’ and their country of origin’s suicide rates [3,9–14]. Another group of studies found that the suicide rates of foreign nationals converge to the suicide rate of the native population [15,16].

This study sought to uncover the patterns in suicides by persons with of foreign background in Japan. To our knowledge, there has been only one systematic study, conducted in the 1980s, that investigated the suicide rates of non-Japanese citizens in Japan [17]. Such a dearth of research is unfortunate, because studying the suicide rates of non-Japanese citizens in Japan is important for at least two reasons. First, Japan is a country that has one of the highest suicide rates in the world and studying the suicide mortality of foreign nationals can help us understand whether their suicide rates tend to “converge” to the suicide rate of the country where they currently reside.

Second, the suicide rates of persons with foreign background may indicate their overall well-being and health in the country of residence. The number of foreign nationals in Japan has increased nearly three-fold since 1980 [18,19]. However, such a rapid increase has also brought a backlash against non-Japanese citizens among a subset of the Japanese population. Hate crime incidents, especially hate speech demonstrations against Koreans, have been increasing in Japan [20]. In addition, non-Japanese citizens, including long-term residents, face a various types of discrimination in areas of housing, employment, and marriage, to name a few [21]. Thus, the conditions that foreign nationals face in Japan are not necessarily positive.

The well-being of Koreans in Japan is of particular interest, as they have a unique historical background. Many Koreans in Japan are not recent immigrants, but long-term residents who were born and raised in Japan. They typically have “special permanent resident status,” which is different from the conventional status permitted to foreigners seeking permanent residence in Japan; the former is granted to those who migrated to Japan when the Korean Peninsula was under Japan’s colonial rule. Although the Koreans under Japanese colonial rule had been considered Japanese citizens during the occupation period, they lost their Japanese citizenship and associated privileges after the War. The special permanent resident status was created in 1991 for them and their descendants to recognize the special historical circumstances surrounding them. As of 2016, 66.1% of Koreans residing in Japan are special permanent residents [22].

Using large-scale data from the national death registry in Japan, this study reported the age-standardized suicide rates of foreign nationals in Japan by sex from 1980 to the mid-2010s. We also computed rate ratios to compare their suicide rates to those of the Japanese population. In addition, we focused on Koreans who have the highest suicide rate in Japan and compared the suicide rates of Koreans in Japan with Koreans in South Korea to examine whether their suicide rates were more closely related to those of their country of origin or those of their host country.

## Data and method

The individual death records in this study were obtained from the Vital Statistics of Japan, which lists all reported deaths in Japan. The Vital Statistics data were collected for administrative purposes by the Ministry of Health, Labour, and Welfare of Japan and were anonymized and de-identified by the Ministry prior to analysis. Individual death records between 1980 and 2014 were made available for research purposes with approval from the Ministry.

The data in the Vital Statistics Report are based on death certificates issued by physicians and are subsequently reported to local governments where the residency of the deceased is registered. The data include the nationality of the deceased, the date of death, and the underlying cause of death based on the International Classification of Diseases (ICD) 8/9 standard (up to 1994) and the ICD-10 standard (1995 to present). Foreigners who are in Japan for a short period, such as travelers, do not appear in the death record. This study analyzed deaths by suicide (ICD-8/9: E950-E959, ICD-10: X60-X84) that occurred in Japan.

Using the Vital Statistics data, we first computed the number of suicides by sex and country of origin. As of 1980, the countries of origin reported in the Vital Statistics were Japan, Korea, China, the United States, and “others.” Five other countries of origin were added in 1992 (the Philippines, Thailand, the United Kingdom, Brazil, and Peru) to be listed separately from the “others” category. Accordingly, we computed the number of suicides for four countries of origin from 1980 to 1991 (Japan, Korea, China, and the United States) and nine countries of origin from 1992 to 2014 (Japan, Korea, China, the United states, the Philippines, Thailand, the United Kingdom, Brazil, and Peru). Because the annual number of suicides among non-Japanese groups can be small, we aggregated the number of suicides over five-year periods (e.g., 1980–1984 and 2010–2014) for each nationality. Note that the number of suicide deaths by those from Brazil, Thailand, the Philippines, Peru, and the United Kingdom were aggregated over three years (1992–1994) for the period 1990–1994 instead of five for the reason described above.

Next, we calculated the age-standardized suicide rates per 100,000 by sex and nationality. We used the population counts by sex, nationality, and five-year age groups from the Population Census of Japan. Regardless of nationality, those who had lived or were going to live in Japan for three months or longer as of the census date were included in the tabulation [23]. The Population Census was conducted every five years, and we used the latest available Census population data for years between Census years. For instance, we used the census population from 1990 to compute the suicide rates for 1990, 1991, 1992, 1993 and 1994.

The suicide rates by sex and nationality were age-standardized because the age composition of the Japanese and non-Japanese population can be different. The standardization was based on the WHO World Standard Population [24]. We report the average of the age-standardized suicide rates over the five-year periods. Using the standardized suicide rates per 100,000, we also calculated the rate ratios and their confidence intervals for the Korean and Chinese population, with the suicide rate of Japanese as the reference. The rate ratios were not calculated for other nationalities as the number of suicide counts in each category was too small.

Our final analysis compared the suicide rates in Japanese and Korean populations in Japan with those of Koreans in South Korea. We calculated the age-standardized suicide rates in South Korea in the same manner, using data on the annual number of suicides by five-year age groups between 1983 and 2014 and the population counts by age group between 1980 and 2010. The suicide and population data were obtained from Statistics Korea and Korea’s quinquennial Population Census, respectively.

## Results

We first present the number of non-Japanese citizens in Japan by eight nationality groups (Table 1). During 1980–2015, the number of foreign nationals increased by more than 160%, with the largest increase observed among Chinese. As of 2015, Chinese and Koreans were the two largest non-Japanese groups. Koreans were the largest group at the beginning of the study period, but by 2010 they were outnumbered by Chinese. During 1980–2015, 263,628 Koreans naturalized [25] as Japanese citizens, which may partly explain their apparent population decrease.

**Table 1:** Population of foreign nationals in Japan: 1980–2015.

	Total Foreign Pop.	Korea	%	China	%	USA	%	Brazil	%	Thailand	%	Philippines	%	Peru	%	UK	%
1980	668675	400530	0.45	34779	0.04	14373	0.02										
1985	720093	427911	0.45	50317	0.05	19792	0.02										
1990	886397	457681	0.45	97152	0.10	28224	0.03	40613	0.04	7226	0.01	34729	0.03	5855	0.01	5622	0.01
1995	1140326	474480	0.45	155400	0.15	33032	0.03	119118	0.11	19948	0.02	64498	0.06	23448	0.02	7970	0.01
2000	1310545	466948	0.43	227046	0.21	32428	0.03	157724	0.15	22445	0.02	87345	0.08	27100	0.03	9171	0.01
2005	1555505	422441	0.39	322833	0.29	32325	0.03	178460	0.16	24860	0.02	114619	0.10	32022	0.03	8871	0.01
2010	1648037	391335	0.36	425547	0.39	33275	0.03	123535	0.11	28133	0.03	132653	0.12	29128	0.03	9225	0.01
2015	1752368	353030	0.32	458823	0.42	37643	0.03	102761	0.09	32479	0.03	155823	0.14	28040	0.03	10494	0.01

Source: Population Census of Japan, 1980–2015.

Note: Brazil, Thailand, the Philippines, Peru, and the United Kingdom were not independently reported until the 1990 census. % indicates the percentage of each population relative to the total population of Japan.

Table 2 presents the total number of suicide deaths aggregated over five-year periods, by sex and nationality. The highest number of suicides among foreign nationals was observed among Koreans for both sexes throughout the study period. The number of suicides sharply increased between the periods 1995–1999 and 2000–2004 in the aftermath of the Asian Financial Crisis, especially among Japanese and Korean males. By 2010–2014, the total suicide counts declined for both Japanese and Korean males. Over the same period, the number of suicides among Japanese females also decreased. However, the number of suicides among Korean females in Japan remained high after 2010, even though their population in Japan had decreased.

**Table 2:** Total number of suicides in Japan over Five-year periods by sex and nationality.

**Male**									
Years	Japan	Korea	China	USA	Brazil	Thailand	Philippines	Peru	UK
1980–1984	71939	553	26	8					
1985–1989	74428	543	23	7					
1990–1994	66016	521	57	11	14	13	4	0	1
1995–1999	89933	757	47	15	34	7	4	5	6
2000–2004	110158	846	65	18	31	4	3	4	4
2005–2009	109691	828	66	14	32	4	5	3	8
2010–2014	94511	587	76	21	33	2	12	3	3
**Female**									
Years	Japan	Korea	China	USA	Brazil	Thailand	Philippines	Peru	UK
1980–1984	38803	181	19	3					
1985–1989	42351	233	32	4					
1990–1994	36347	241	23	3	5	10	5	0	2
1995–1999	40435	301	50	10	9	15	7	1	1
2000–2004	42127	323	62	5	5	12	13	0	1
2005–2009	42810	345	70	3	21	12	20	1	2
2010–2014	40832	378	87	5	6	14	21	1	0

Source: Vital Statistics of Japan, 1980–2014.

Note: The total number of suicides aggregated over five-year periods are reported. The 1990–1994 data for Brazil, Thailand, the Philippines, Peru, and the United Kingdom were aggregated over three years (1992–1994).

Table 3 reports the age-standardized suicide rates per 100,000 for residents in Japan by nationality to facilitate comparisons across nationalities. Except for the Japanese, the table also shows the age-standardized suicide rates in each group’s country of origin in parentheses. The data are available only for 2000, 2010, and 2015 [26]. According to Table 3, the suicide rates of Koreans living in Japan were by far the highest among all nationalities for both sexes. For example, during the 2005–2009 period, the suicide rates for Korean males and females in Japan were 73.64 and 27.31 per 100,000, respectively, which were approximately twice as high as the suicide rates of their Japanese counterparts (37.65 and 13.49). The suicide rate of Korean females rose since the 1995–1999 period, while the suicide rates of other groups had declined by the 2010–2014 period. The suicide rates of Chinese residents decreased from 28.53 in 1980 to 12.54 by the latest period in our study. Comparison of the suicide rates of non-Japanese citizens in Japan and rates of their country of origin reveals that for most nationalities, the suicide rates of residents in Japan tended to resemble those in the home country. However, there were some exceptions; notably Korean and Chinese males and Korean females in Japan had higher suicide rates compared to those in their home country.

**Table 3:** Standardized suicide rates by sex and nationality, 1980–2014.

*Male*									
***Years***	**Japan**	**Korea**	**China**	**USA**	**Brazil**	**Thailand**	**Philippines**	**Peru**	**UK**
*1980–1984*	33.59	58.64	28.53	21.31					
*1985–1989*	31.59	53.99	19.68	13.68					
*1990–1994*	25.78	47.28	30.96	17.04	13.20	61.24	10.84	0.00	9.25
*1995–1999*	32.34	65.14	15.90	16.15	8.00	10.54	4.84	4.90	55.40
*2000–2004*	37.76	70.33	17.91	18.18	6.30	13.98	14.46	5.29	12.85
		(20.8)	(12.7)	(16.5)	(8.9)	(23.7)	(4.5)	(8.8)	(12.7)
*2005–2009*	37.65	73.64	16.39	11.19	7.52	9.58	5.20	3.78	19.44
		(39.3)	(9.0)	(18.6)	(9.0)	(21.0)	(5.7)	(7.5)	(10.6)
*2010–2014*	33.28	56.74	12.54	16.30	9.74	6.21	7.66	3.07	7.46
		(31.4)	(7.9)	(20.5)	(9.7)	(21.7)	(5.4)	(7.6)	(11.6)
Female									
***Years***	**Japan**	**Korea**	**China**	**USA**	**Brazil**	**Thailand**	**Philippines**	**Peru**	**UK**
*1980–1984*	15.89	20.22	23.61	7.77					
*1985–1989*	15.48	22.87	23.92	8.65					
*1990–1994*	11.98	20.87	11.62	10.59	9.77	35.35	9.76	0.00	60.34
*1995–1999*	12.54	23.55	15.20	17.74	3.38	17.63	1.32	1.02	8.43
*2000–2004*	12.80	23.50	11.47	10.55	1.16	16.54	9.74	0.00	2.78
		(8.5)	(15.6)	(4.1)	(2.2)	(8.3)	(1.8)	(3.3)	(3.8)
*2005–2009*	13.49	27.31	11.54	5.20	4.33	8.50	4.54	0.88	10.13
		(18.7)	(11)	(5.2)	(2.8)	(5.5)	(2.3)	(2.5)	(3.2)
*2010–2014*	12.89	29.88	9.40	10.10	1.88	6.01	5.24	1.88	0.00
		(12.5)	(8.5)	(6.3)	(2.9)	(4.9)	(2.3)	(2.7)	(3.7)

Source: Vital Statistics of Japan, 1980*–*2014, Population Census of Japan, 1980*–*2010 (for data on Japanese residents). World Health Organization (data in home countries) [26].

Note: Age-standardized suicide rates aggregated over five-year periods are reported. The 1990*–*1994 data for Brazil, Thailand, the Philippines, Peru, and the United Kingdom were aggregated over three years (1992*–*1994). The numbers in parentheses report the age-standardized suicide rates in the country of origin. The data were available only from 2000, and the numbers in 2000, 2010, and 2015 are shown.

**Fig 1:**
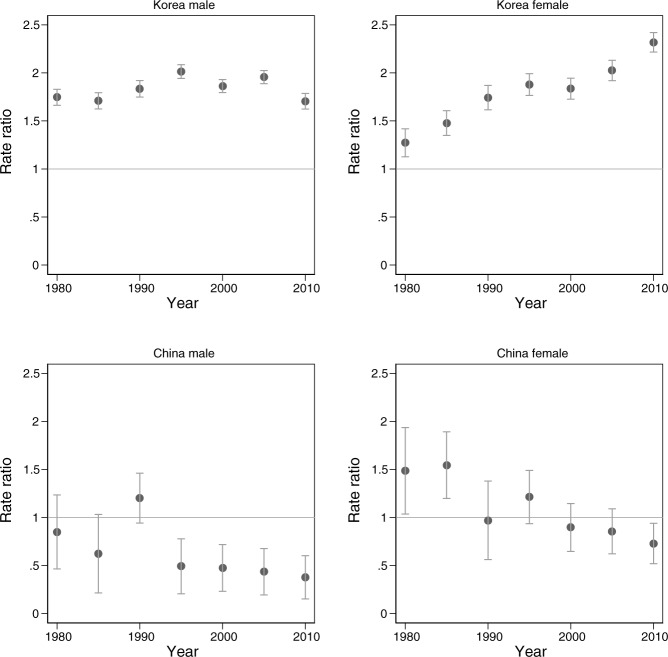
The Rate ratios of standardized suicide rates. Source: Vital Statistics of Japan, 1980–2014, Population Census of Japan, 1980–2010. Note: The rate ratios were calculated with the suicide rate of Japanese citizens as a reference.

Fig 1 presents the rate ratios (RRs) of the suicide rates for the Korean and Chinese population by sex, with Japanese suicide rates as the reference group. The vertical bars represent 95% confidence intervals. According to the top panel, which depicts the RRs for Koreans, the suicide rates of Korean males in Japan were more than 50% higher than those of Japanese males throughout the study period. In the 1995–1999 period, the RR was 2.014 (95% CI: 1.943–2.086), which suggests that Korean males in Japan were twice as likely to die by suicide compared to Japanese males. Regarding Korean females, at the beginning of the study period, they were approximately 25% more likely to die by suicide than Japanese females, but the ratio gradually increased over time. By the 2010–2014 period, the RR reached 2.318 (95% CI: 2.217–2.419), which indicates that the suicide rate among Korean females was c. 130% higher than that of Japanese females by the latest study period.

We found different patterns among the Chinese population in Japan. According to the bottom panel of Fig 1, the suicide rates of Chinese males were similar to those of Japanese males until 1990–1994 but became significantly lower after 1995. The RRs for Chinese females also statistically did not differ from a value of one, except for the first two periods, in which their suicide rates were approximately 50% higher than those of Japanese females.

**Fig 2:**
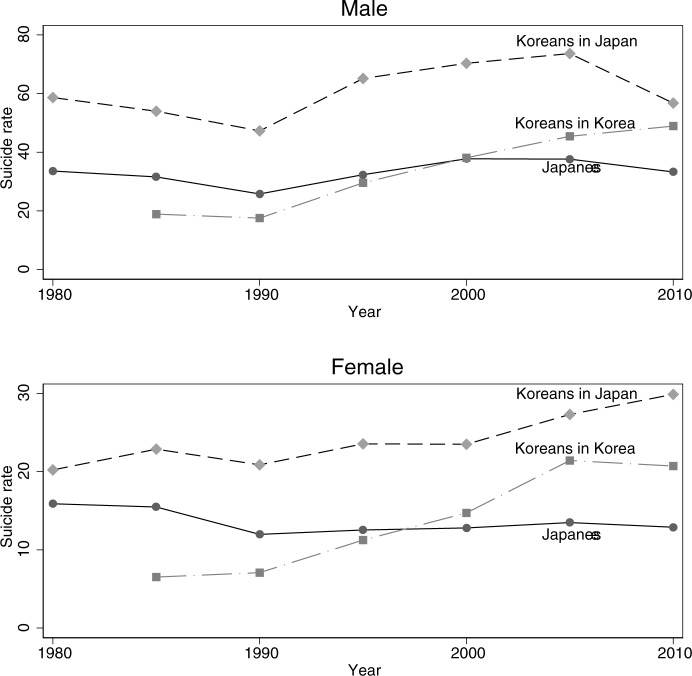
Trends in standardized suicide rates by sex and nationality and the country of residence. Source: Vital Statistics of Japan, 1980–2014, Population Census of Japan, 1980–2010, Statistics Korea, 1980–2014. Notes: The age-standardized suicide rates aggregated over five-year periods reported.

Finally, we compared the trends in the suicide rates for Koreans living in Japan, Koreans living in South Korea, and Japanese citizens, to determine whether the suicide rates of Koreans living in Japan were more closely related to the rates of Koreans in South Korea, which may arguably be considered their country of origin, or to the suicide rates of Japanese citizens. As shown in the top panel of Fig 2, the suicide rates of Korean males in Japan followed a similar trend to that of Japanese males. The suicide rates of Korean females in Japan and Japanese females in Japan also exhibited similar trends, but the difference between their rates increased toward the end of our study period. In contrast, the suicide rates of Korean females in Japan and those in South Korea became more similar in recent years. Finally, Korean males and females residing in Japan had higher suicide rates than Koreans in South Korea throughout the study period. However, the gap between Koreans in Japan and Koreans in South Korea narrowed in recent years.

## Discussion

The present study’s contribution is that it examined long-term trends in suicide deaths among foreign nationals in Japan from eight countries of origin. This is the first study that provides comprehensive data regarding suicides by non-Japanese citizens in Japan using large-scale data that include all reported deaths in Japan.

We found that the suicide rates of non-Japanese citizens in Japan differ across nationalities. The suicide rates of Koreans and Chinese in Japan were similar to or higher than those of Japanese, while other groups tended to show lower suicide rates. This suggests that immigrants in Japan are not necessarily influenced by the suicide rates of the host country (i.e., Japan). For example, the suicide rate of the Japanese population sharply increased in the mid-1990s, but such an increase was not observed among foreign nationals living in Japan. The results reported in Table 3 suggest that immigrant suicide rates are thus more likely to be influenced by other factors, such as the suicide rates of their country of origin [3, 9–14]. However, this result should be interpreted with caution because the size of immigrant population in Japan is small and suicide is a rare event, as shown in Tables 1 and 2. As a result, the suicide rates tend to vary considerably over years, which makes it challenging to compare across nationalities or over time.

We also found that the Korean population in Japan had consistently high suicide rates from 1980 to the mid-2010s, which were nearly twice as high as those of the Japanese population. Both Korean males and females exhibit high suicide rates, and the suicide rate of Korean females has been increasing over time. While the length of their residency in Japan may explain why the suicide rates of Koreans in Japan tended to follow a similar temporal pattern to those of the Japanese population, it does not explain why their suicide rates were considerably higher than the suicide rates of Japanese and Koreans in South Korea. There could be multiple factors underlying their relatively high suicide rates, but of particular relevance is that Korean residents tend to face various disadvantages and adversities in Japan.

As noted above, many Koreans in Japan are special permanent residents, and the younger generation was born and educated in Japan with Japanese as their native language. However, their socioeconomic status is not equivalent to that of the Japanese native population. According to the Population Census of Japan in 2010, the unemployment rate of Korean males aged 15 or higher was 4.6 percentage points higher than that of Japanese males. Even among those aged 25–44, the difference was 4.0 percentage points [19]. The same census data also indicate that Japanese and Korean males engage in different types of work: 61% of Japanese males work as full-time office workers, whereas only 41% of Korean males work as such.

In addition, long-term Korean residents in Japan face discrimination and hardship to date. For example, according to a survey administered at the request of the Ministry of Justice, 27.2% of respondents with a special permanent resident status answered that a housing application had been denied within the prior five years due to their nationality [21]. Note that nearly all (99%) special permanent residents are Koreans [22]. In the same survey, 16.1% of special permanent residents responded that their romantic relationship with a Japanese partner was opposed by the partner’s Japanese relatives because of the respondent’s nationality. Some Koreans also experience hardship at school and in the workplace: 19.3% of special permanent residents answered that their relationships with others at school or in the workplace were negatively affected because their Japanese peers are prejudiced against foreigners, and 10.1% of respondents reported that they were bullied at school or in the workplace because of their nationality.

Finally, the number of hate speech and street demonstrations against Koreans and Chinese in Japan has been increasing throughout Japan. A total of 378 such street rallies were recorded in 2014, some of which contained strong racist speech toward long-term Korean residents [20]. The spread of hate speech and rallies against foreigners was mentioned as a source of concern in a country report by the United Nations [27].

In summary, circumstances facing Koreans living in Japan can be challenging. Despite the fact that many Korean residents in Japan have integrated with the local language and culture, their aggregate suicide rate is significantly higher than the suicide rate among Japanese citizens. Although we cannot immediately conclude that these high suicide rates are due to the conditions that Koreans face in Japan, it is possible that such conditions influence the decisions of some Koreans to kill themselves. Understanding the causes underlying their high suicide rates is an important future research agenda.

Several limitations of this study should be noted. First, our analysis did not consider length of stay in Japan as it was not recorded in the Vital Statistics. As discussed above, Koreans in Japan can be short-term residents, such as college students, or long-term special permanent residents. We expect that suicide rates would vary depending on length of stay, but we could not assess this in the present analysis. Second, the census population that we used to calculate the standardized suicide rates may not be accurate. The census count only reflected population as of the census date and undercounting foreign nationals was also a possibility. Third, our analysis did not consider the role of socioeconomic status such as income and employment. For example, Koreans in Japan might exhibit higher suicide rates than Japanese because the former might be more likely than the latter to be unemployed.

Despite these limitations, the present study provides important data regarding suicide rates among foreign nationals in Japan. Our study also has significant policy implications. The findings of this study strongly suggest that extra care should be taken to ensure the well-being of foreign nationals, even if they live in a host country for a sufficient period of time. As the high suicide rates of Koreans in Japan documented in this study indicate, language acquisition and acculturation are insufficient to ensure the well-being and health of foreign nationals. Their risk of suicide may remain high until the host country fully accepts them as members of society.
